# Correction: *Pichia pastoris* growth—coupled heme biosynthesis analysis using metabolic modelling

**DOI:** 10.1038/s41598-025-20866-1

**Published:** 2025-09-30

**Authors:** Agris Pentjuss, Emils Bolmanis, Anastasija Suleiko, Elina Didrihsone, Arturs Suleiko, Konstantins Dubencovs, Janis Liepins, Andris Kazaks, Juris Vanags

**Affiliations:** 1 Microbiology and Biotechnology Institute, University of Latvia, Jelgavas Street 1, Riga, 1004 Latvia; 2 Latvian State Institute of Wood Chemistry, Dzerbenes Street 27, Riga, 1006 Latvia; 3 Latvian Biomedical Research and Study Centre, Ratsupites Street 1 K-1, Riga, 1067 Latvia; 4 Bioreactors.Net AS, Dzerbenes Street 27, Riga, 1006 Latvia

Correction to: *Scientific Reports* 10.1038/s41598-023-42865-w, published online 22 September 2023

The Supplementary Information 1 file published with this Article contained an error where an incomplete file was uploaded. The original Supplementary Information 1 file is provided below.

This error has now been corrected in the Supplementary Information 1 file that accompanies the original Article.

## Supplementary Information

Supplementary Information 1.

