# Supplementary material for: Pichia pastoris growth—coupled heme biosynthesis analysis using metabolic modelling
Source: Sci Rep. 2023 Sep 22;13:15816. doi: 10.1038/s41598-023-42865-w (PMC10516909; doi:10.1038/s41598-023-42865-w)
Supplement: Supplementary file 2 — Supplementary Information 2. [file 41598_2023_42865_MOESM2_ESM.docx]

**Supplementary materials 1**

**CER, OUR and cell biomass estimation.**

Carbon evolution rate (CER) and oxygen uptake rate (OUR) were estimated using the O_2_/CO_2_ analyzer and calculated using the following formulas:

| $CER= \frac{F_{air}*P}{V*R*T}*\left( \frac{1-O_{2}\%in-{CO}_{2}\%in}{1-O_{2}\%out-{CO}_{2}\%out}*{CO}_{2}\%out-{CO}_{2}\%in \right)$ | (1) |
| --- | --- |
| $OUR= \frac{F_{air}*P}{V*R*T}*\left( O_{2}\%in-\frac{1-O_{2}\%in-{CO}_{2}\%in}{1-O_{2}\%out-{CO}_{2}\%out}*O_{2}\%out \right)$ | (2) |

where, Fair – inlet air flow (L/h), P – normal pressure (1.0133 bar), V – bioreactor working volume (L), R – gas constant (8.314*10-2 bar L K-1 mol-1), T – process temperature (K), O_2_%in and O_2_%out – oxygen concentration in the inlet and outlet gas, respectively (%), CO_2_%in and CO_2_%out – carbon dioxide concentration in the inlet and outlet gas, respectively (%).

Cell biomass concentration was estimated using the Optek (turbidity) and Incyte (permittivity) sensor probes. Correlations between sensor signals and cell biomass were established using the experimental measurements as reference.

The Incyte sensor measures culture dielectric permittivity, and, thus, correlates only with the viable cell fraction in the bioreactor, which is preferable for metabolic modelling, as reactions can happen only in viable cells. As the experimentally measured cell concentrations include also the dead cell fraction, an empirical cell death rate coefficient (µd = 0.033 h-1) can be estimated using the sensor data. This coefficient can further be applied to the turbidity results to correlate the signal with the alive cell fraction.

**Table S1.** Dry cell biomass (X_dry_), CER, OUR and specific growth rate estimations, based on Optek (turbidity) and Incyte (permittivity) sensor measurements at the end of each cultivation phase.

|  | **X_dry_ (g/L)** | **CER (mmol g^-1^ h^-1^)** | **OUR (mmol g^-1^ h^-1^)** | **µ (h^-1^)** |
| --- | --- | --- | --- | --- |
| *Optek sensor measurement* | | | | |
| Glycerol | 62.5 | 1.61 | 2.17 | 0.19 |
| Methanol | 89.0 | 1.13 | 1.76 | 0.03 |
| *Incyte sensor measurement* | | | | |
| Glycerol | 59.2 | 1.70 | 2.29 | 0.19 |
| Methanol | 110.85 | 0.91 | 1.41 | 0.04 |

**Supplementary materials 2**

**Optimisation “Optimisation_result_Final” MS Excel file Content**

1. “S6.1.All_in_one” – all *P. pastoris* iMT1026 genome-scale metabolic model reactions.
2. “S6.2.Dependant_reactions” – all reactions which have an impact on ferroheme *b production.*
3. “S6.3.Pos_contra_proportional”- reactions with flux value from left to right and are inversely proportionally to forced product (heme) changing fluxes;
4. “S6.4.Neg_contra_proportional” - reactions with flux value from right to left and are inversely proportionally to forced product (heme) changing fluxes;
5. “S6.5.Pos_directly_p roportional” - reactions with flux value from left to right and are directly proportionally to forced product (heme) changing fluxes;
6. “S6.6.Neg_directly_proportional” - reactions with flux value from right to left and are directly proportionally to forced product (heme) changing fluxes.
7. “S6.7.Directly_proportional” – All reactions which are directly proportional. Excluded all transport reactions, reactions with less than 5 flux values, and inconsistent flux values.
8. “S6.8.Inversely proprotional” – All reactions which are directly proportional. Excluded all transport reactions, reactions with less than 5 flux values, and inconsistent flux values.
9. “S6.9.Inversly_reactions” – Best none amino acids downregulation candidates and graphical comparision of reaction involvement in specific biochemical pathways.
10. “S6.10. Inversly_amino” - Best amino acids downregulation candidates and graphical comparision of reaction involvement in specific biochemical pathways.
11. “S6.11.downr_other_reac_sugest” – detailed information of best none amino acids downregulation candidates.
12. “S6.12.downr_amino_reac_sugest” - detailed information of best amino acids downregulation candidates.
13. “S6.13_meth_glyc_exp_vs_FBA” – Comparison of Optek and Incyte experimental data with GSM optimisation results. CO2, O2 and substrate (Glycerol or Methanol) data were used to constraint GSM.

**Supplementary materials 3**

**Upregulation reaction candidates and their description.**

All reactions in the supplementary material will be described as:

Reaction name (ID in the model) (ID in the Metacyc) (Found in Supplementary material table).

All upregulation biosynthesis pathways are found in S6.7 Supplementary materials.

Text in green represents specific reaction E.C number and in the graphics shows exact place in biochemical pathway.

**Formaldehyde assimilation III (dihydroxyacetone cycle) (P185-PWY) reactions**

2.2.1.3 - Dihydroxyacetone synthase (DAS) (FORMALDEHYDE-TRANSKETOLASE-RXN) (S6.7 ID 26);

2.7.1.29 - Dihydroxyacetone kinase – (DHAKx) (GLYCERONE-KINASE-RXN) (S6.7 ID 27).

These reactions are closely interconnected and allow methylotrophs to metabolise methanol (meoh) (METOH) to formaldehyde (fald) (FORMALDEHYDE), which later forms Glyceraldehyde 3-phosphate (G3P) (GAP) and dihydroxyacetone (DHA) (DIHYDROXYACETONE) - the building block chemicals for all other metabolic processes, including of heme biosynthesis.


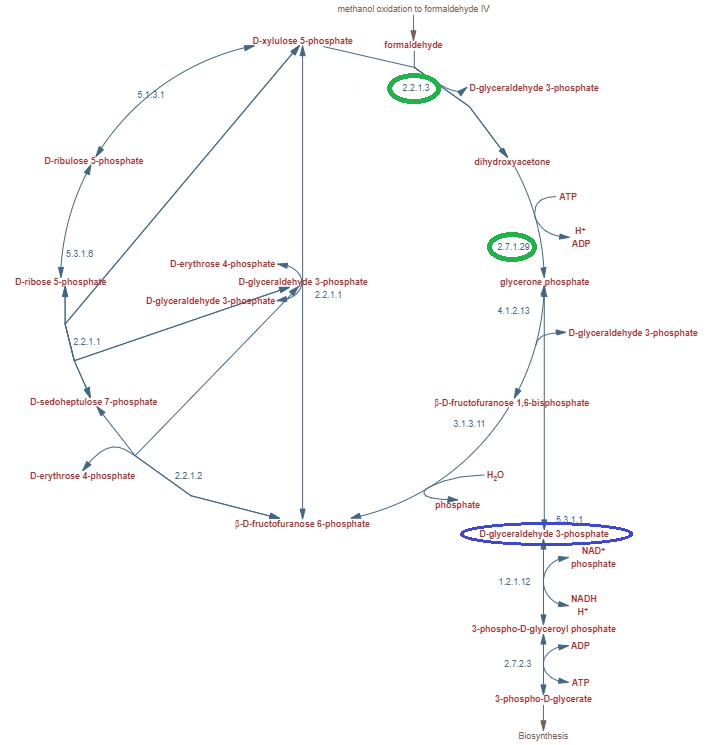


**Figure S3.1.** Dihydroxyacetone cycle metabolic reactions graphic. Rebuild using Metacyc graphics (P185-PWY). Green cirlces show upregulation candidate reactions

**Folate transformations I (PWY-2201)**

**1.5.1.6** - Formyltetrahydrofolate (FTHFDH) (FORMYLTETRAHYDROFOLATE-DEHYDROGENASE-RXN) (S6.7 ID 1);

**1.5.1.5** – Methylenetetrahydrofolate dehydrogenase (NADP) (MTHFD) (METHYLENETHFDEHYDROG-NADP-RXN) (S6.7 ID 12);

**2.1.2.1** - Glycine hydroxymethyltransferase (GHMT2r) (GLYOHMETRANS-RXN) (S6.7 ID 9).

Formyltetrahydrofolate dehydrogenase (FTHFDH) (FORMYLTETRAHYDROFOLATE-DEHYDROGENASE-RXN) and methylenetetrahydrofolate dehydrogenase (NADP) (MTHFD, which are one-carbon metabolism pathway reactions (Fig S3.2). responsible for the interconversion of different tetrahydrofolate (THF) forms.

Glycine hydroxymethyltransferase (GHMT2r) is also involved in one-carbon metabolism and in anaerobic purine degradation processes.


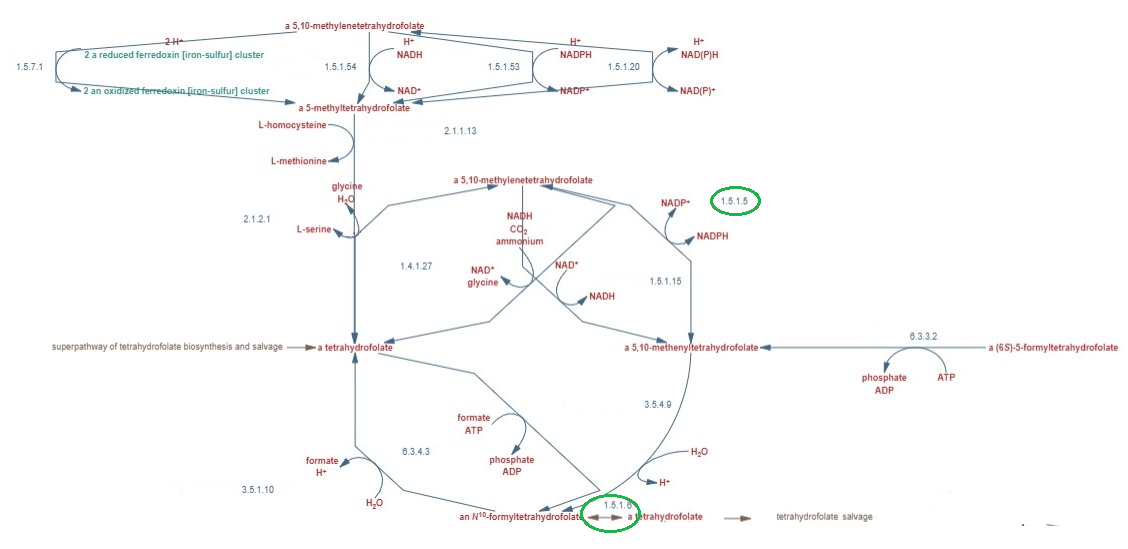


**Figure S3.2.** Folate transformations metabolic reactions graphic. Rebuild using Metacyc graphics (PWY-2201). Green cirlces show upregulation candidate reactions. Purple circles show in the text described building block chemicals.

**L-serine biosynthesis I (SERSYN-PWY)**

1.1.1.95 - Phosphoglycerate dehydrogenase (PGCD) (PGLYCDEHYDROG-RXN) (S6.7 ID 9)

2.6.1.52 - phosphoserine transaminase (PSERT) (PSERTRANSAM-RXN) (S6.7 ID 17).

3.1.3.3 - phosphoserine phosphatase (L-serine) (PSP_L) (RXN0-5114) (S6.7 ID 18).


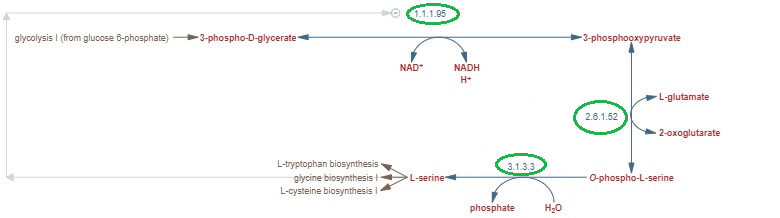


**Figure S3.3.** L-serine biosynthesis metabolic reactions graphic. Rebuild using Metacyc graphics (SERSYN-PWY). Green cirlces show upregulation candidate reactions

Phosphoglycerate dehydrogenase (PGCD), phosphoserine transaminase (PSERT), phosphoserine phosphatase (L-serine) (PSP_L) are serine biosynthesis reactions (Fig.S3.3), which leads to glycine (gly) (GLY) biosynthesis. Glycine (gly) (GLY) is the precursor of the tetrapyrrole biosynthesis pathway (PWY-5189).

**Glutamate dehydrogenase (GLUTDEHYD-RXN).**

1.4.1.4 - glutamate dehydrogenase (NADP^+^) (GLUDyi) (GLUTDEHYD-RXN) (S6.7 ID 10)

Glutamate dehydrogenase (GLUDyi) (GLUTDEHYD-RXN) and pyruvate carboxylase ( PCH ) (PYRUVATE-CARBOXYLASE-RXN) are glycine (gly) (GLY) biosynthesis precursors. Also, glycine is the precursor of heme-BIOSYNTHESIS-II (heme-BIOSYNTHESIS-II) and tetrapyrrole biosynthesis (PWY-5189) pathways which produce heme.

**Pyruvate carboxylase (PYRUVATE-CARBOXYLASE-RXN)**

6.4.1.1 - pyruvate carboxylase (PC) (PYRUVATE-CARBOXYLASE-RXN) (S6.7 ID 13).

Oxaloacetate and 2-oxoglutarate are involved in oxoglutarate-isocitrate shuttle, which regulates NADPH redox shuttle between mitochondria and cytosol ^1^

The conversion of glutamate (glu_L) (GLT) to proline (pro_L) (PRO) and *vice versa* is thought to play a role in ATP (atp) (ATP) increase. This could be related to 5-aminolevulinate synthase (ALASm) (5-AMINOLEVULINIC-ACID-SYNTHASE-RXN), where Succinyl CoA (succoa) is consumed in large amounts as a 5-Aminolevulinate (5aop) (5-AMINO-LEVULINATE) precursor. In the mitochondrial TCA cycle (PWY-5690) (Fig. S3.4) Succinate--CoA ligase (SUCCCOASYN-RXN) (6.2.1.5) reaction, which is one of the suggested upregulation reactions is used to produce Succinyl CoA (SucCoA) (SUC-COA) and consume large amounts of ATP (atp) (ATP), which could be a limiting step for improved intracellular heme production if less ATP atp) (ATP) is available in mitochondria.


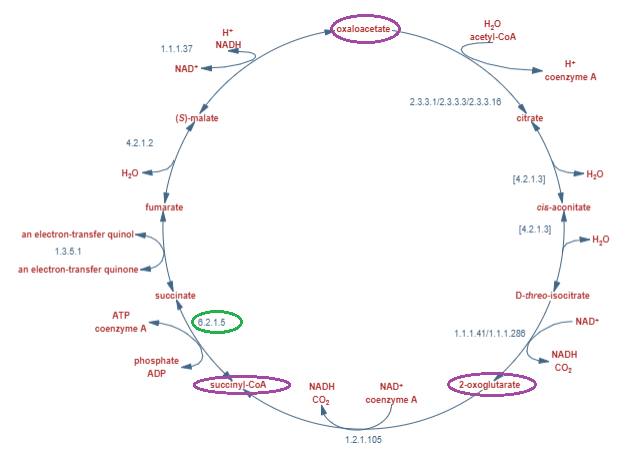


**Figure S3.4**. TCA cycle II metabolic reactions graphic. Rebuild using Metacyc graphics (PWY-5690). Green cirlces show upregulation candidate reactions. Purple circles show in the text described building block chemicals.

**Tetrapyrrole biosynthesis II (from glycine) (PWY-5189)**

2.3.1.37 – 5-aminolevulinate synthase (ALASm) (5-AMINOLEVULINIC-ACID-SYNTHASE-RXN) (S6.7 ID 13).

4.2.1.24 - porphobilinogen synthase (PPBNGS) (PORPHOBILSYNTH-RXN) (S6.7 ID 15).

2.5.1.61 - hydroxymethylbilane synthase (HMBS) (OHMETHYLBILANESYN-RXN) (S6.7 ID 11).

4.2.1.75 - uroporphyrinogen-III synthase (UPP3S) ( UROGENIIISYN-RXN) (S6.7 ID 24).


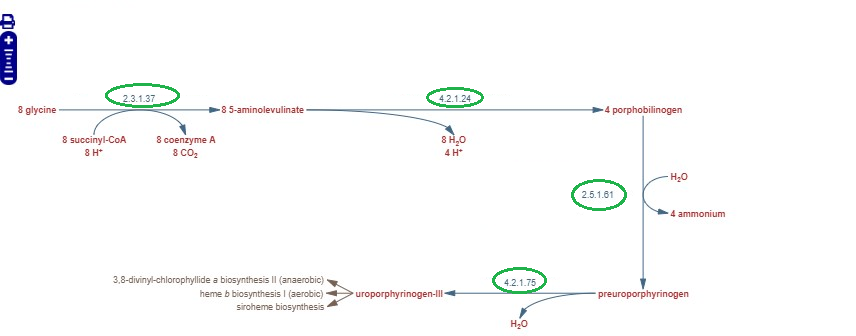


**Figure S3.5**. Tetrapyrrole biosynthesis metabolic reactions in the graphic. Rebuild using Metacyc graphics (PWY-5189). Green cirlces show upregulation candidate reactions.

To increase intracellular heme bioproduction the most perspective upregulation reactions are 5-aminolevulinate synthase (ALASm) (5-AMINOLEVULINIC-ACID-SYNTHASE-RXN), porphobilinogen synthase (PPBNGS) (PORPHOBILSYNTH-RXN), hydroxymethylbilane synthase (HMBS) (OHMETHYLBILANESYN-RXN), uroporphyrinogen-III synthase (UPP3S) UROGENIIISYN-RXN from tetrapyrrole biosynthesis II (from glycine) (PWY-5189) (Fig. S3.5). In this pathway is produced uroporphyrinogen-III (uppg3) (UROPORPHYRINOGEN-III), which is the precursor of the heme biosynthesis pathway.

**Heme b biosynthesis I pathway (heme-BIOSYNTHESIS-II).**

4.1.1.37 - uroporphyrinogen decarboxylase (UPPDC1) (UROGENDECARBOX-RXN) (S6.7 ID 25)

1.3.3.3 - coproporphyrinogen oxidase (CPPPGO) (RXN0-1461) (S6.7 ID 4).

1.3.3.4 - protoporphyrinogen oxidase (PPPGOm) (PROTOPORGENOXI-RXN) (S6.7 ID 16).

4.98.1.1 - protoporphyrin ferrochelatase (FCLTm) (PROTOHEMEFERROCHELAT-RXN) (S6.7 ID 7).


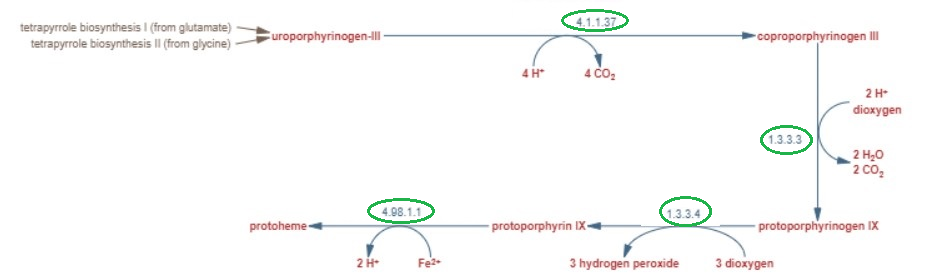
 **Figure S3.6**. Heme b biosynthesis metabolic reactions in the graphic. Rebuild using Metacyc graphics (heme-BIOSYNTHESIS-II). Green cirlces show upregulation candidate reactions.

Uroporphyrinogen decarboxylase (UPPDC1) (UROGENDECARBOX-RXN), coproporphyrinogenase (CPPPGO) (RXN0-1461), protoporphyrinogen oxidase (PPPGOm) (PROTOPORGENOXI-RXN), protoporphyrin ferrochelatase (FCLTm) (PROTOHEMEFERROCHELAT-RXN) are reactions found in heme b biosynthesis I (aerobic) pathway (heme-BIOSYNTHESIS-II) (Fig. S3.6), which is the main biochemical pathways for heme production. Both heme b biosynthesis I (aerobic) and tetrapyrrole biosynthesis II (from glycine) pathways together forms heme biosynthesis metabolic pathway (Porphyrin and Chlorophyll Metabolism in GSM).

**Supplementary materials 4**

**Amino acids downregulation candidates reactions and their description.**

All amino acids downregulation candidates cluster contains 25 reactions, which are found in:

1. Arginine and proline Metabolism;
2. Cysteine metabolism,
3. Glycine and serine metabolism;
4. Methionine metabolism;
5. Threonine and lysine metabolism, tyrosine, tryptophan, phenylalanine metabolism;
6. Valine, leucine, and isoleucine metabolism.

All suggested down-regulation reactions have ~ 17.34 % of step-weighted factor and have an equal impact on heme intracellular production.

All reactions in the supplementary material will be described as:

Reaction name (ID in the model) (ID in the Metacyc) (Found in Supplementary material table)

All amino acids’ downregulation biosynthesis pathways are found in S6.12 supplementary materials

**Arginine and proline metabolism**

This amino acid biosynthesis cluster consists of:

6.3.5.5- Carbamoyl-phosphate synthase (glutamine-hydrolysing) (CBPSn) (CARBPSYN-RXN) (S6.12 ID 1).

The reaction converts glutamine (gln_L) (GLN) to glutamate (glu_L) (glutamate) and is an intermediate for L-arginine (arg_L) (ARG) biosynthesis. Reaction downregulation could free additional resources to increase heme intracellular production.

**Cysteine metabolism.**

Amino acids biosynthesis cluster, which consists of:

2.7.7.5 - Sulfate adenylyltransferase (SLFAT) (SULFATE-ADENYLYLTRANSFERASE-ADP-RXN) (S6.12 ID 2);

4.2.1.22 - L-serine hydro-lyase (CYSTH2SS) (RXN-19038) (S6.12 ID 3);

1.8.1.2 - Sulfite reductase (SULR) (SULFITE-REDUCT-RXN) (S6.12 ID 4).

All three reactions are involved in cysteine biosynthesis and convert sulfate or hydrogen sulphide, which greatly impacts sulfur-containing amino acids biosynthesis ^2^. We assume that downregulating sulfate and hydrogen sulphide biosynthesis it is possible to divert additional resources for heme production.

**Glycine and Serine Metabolism.**

Amino acids biosynthesis cluster, which consists of :

1.1.1.3 - Homoserine dehydrogenase (HSDxi) (HOMOSERDEHYDROG-RXN) (S6.12 ID 5);

The reaction produces homoserine, which is the precursor of methionine amino acid.

**Methionine Metabolism.**

Amino acids biosynthesis cluster, which consists of:

4.2.99.9 (2.5.1.48) O-succinylhomoserine lyase (SHSL1) (CYSPH-RXN) (S6.12 ID 6);

4.4.1.13 - cystathionine b-lyase (CYSTL) (CYSTATHIONINE-BETA-LYASE-RXN) (S6.12 ID 7);

4.3.1.- - O-succinylhomoserine lyase (SHSL4r) (METBALT-RXN) (S6.12 ID 8).

All three reactions are involved in methionine precursors biosynthesis. Methionine is sulphur containing amino acid, it participates in cysteine synthesis and it participates in one carbon (1C) metabolism. Potentially, by downregulating cysteine related metabolism (as aforementioned 3 reactions) - it would be possible to divert more methionine to 1C metabolism and consequently - to heme production ^3^.

**Threonine and Lysine Metabolism**

Amino acids biosynthesis cluster, which consists of:

1.1.1.87 – homoisocitrate dehydrogenase (HICITDm) (RXN-7970) (S6.12 ID 9);

E.C NA - non-enzymatic reaction (OXAGm) (RXN-8167) (S6.12 ID 10);

4.2.1.36 (4.2.1.114) - homoacontinate hydratase (HOMOACONITATE-HYDRATASE-RXN) (S6.12 ID 11);

E.C. N.A. - 2-methylcitrate dehydratase (MCITDm) (RXN3O-1983) (S6.12 ID 12);

1.3.99.7 (1.3.8.6) - glutaryl-CoA dehydrogenase (GLUTCOADHc) (GLUTARYL-COA-DEHYDROGENASE-RXN) (S6.12 ID 13);

1.2.1.105 - 2-oxoadipate dehydrogenase complex (2OXOADOX) (2-KETO-ADIPATE-DEHYDROG-RXN) (S6.12 ID 14).

All six reactions are involved in Threonine and Lysine biosynthesis. Most reactions are involved in lysine and threonine degradation products, such as glutaryl-COA production, which further degradation leads to 2 molecules of acetyl-COA and one molecule CO2. We believe that this amino acid degradation reactions down-regulation could increase heme production by decreasing CO2 by-production (PWY-5177 and LYSINE-DEG1-PWY).

**Tyrosine, Tryptophan, and Phenylalanine Metabolism**

Amino acids biosynthesis cluster, which consists of:

4.1.2.15 (2.5.1.54) - 2-deoxy-D-arabino-heptulosonate 7-phosphate synthetase (DDPAm) (DAHPSYN-RXN) (S6.12 ID 15);

4.2.1.20 - tryptophan synthase (TRPS2) (TRYPSYN-RXN) (S6.12 ID 16);

4.1.2.8 (4.2.1.20) - tryptophan synthase (TRPS3r) (RXN0-2381) (S6.12 ID 17);

2.6.1.1 - phenylalanine transaminase (PHETA1) (RXN-10814) (S6.12 ID 18);

2.6.1.58 - L-phenylalanine:pyruvate aminotransferase (PHEPYRAT) (2.6.1.58-RXN) (S6.12 ID 19).

All five reactions are involved in Tyrosine, Tryptophan, and Phenylalanine Metabolism. TRPS2 and TRPS3r reactions (TRPSYN-PWY) and DDPAm are involved in L-tryptophan amino acid precursors’ bioproduction and interconversion. Reactions downregulation could decrease L-tryptophan production and increase intracellular heme biosynthesis. PHEPYRAT reaction is producing L-alanine (ala_L) (L-ALPHA-ALANINE) from phenylalanine (phe_L) (PHE). All reaction downregulation could increase heme production by decreasing L-tryptophan production.

**Valine, Leucine, and Isoleucine Metabolism**

4.1.3.12(2.3.3.13) - 2-isopropylmalate synthase (IPPSm) (2-ISOPROPYLMALATESYN-RXN) (S6.12 ID 20);

E.C. N.A - acetolactate synthase (ACLSm) (DIHYDROXYISOVALDEHYDRAT-RXN) (S6.12 ID 21);

4.2.1.9 - dihydroxy-acid dehydratase (2,3-dihydroxy-3-methylbutanoate) (DHAD1m) (DIHYDROXYISOVALDEHYDRAT-RXN) (S6.12 ID 22);

1.1.1.85 (1.1.1.-) - (2R,3S)-3-methylmalate: NAD+ oxidoreductase (E3MMALOR) (RXN-7745) (S6.12 ID 23).

4.2.1.35 – (R)-2-Methylmalate hydro-lyase (CITCONH) (R-2-METHYLMALATE-DEHYDRATASE-RXN) (S6.12 ID 24);

E.C. N.A - 3-isopropylmalate/(R)-2-methylmalate dehydratase (E3MMALH) (RXN-7744) (S6.12 ID 25);

2.3.1.182 (2.3.3.21) - D-citramalate synthase (CITMALS) (RXN-7743) (S6.12 ID 26).

E3MMALOR, CITCONH, E3MMALH, and CITMALS reactions are involved in L-isoleucine biosynthesis (PWY-5101) metabolism, where their downregulation could improve heme production by decreasing L-isoleucine (ile_L) (ILE) production from pyruvate (pyr) (pyruvate). IPPSm reaction is involved in L-leucine biosynthesis (LEUSYN-PWY) from L-valine (val_L) (VAL) and reaction downregulation could lead to decreased L-leucine (leu_L) (LEU) biosynthesis thus releasing additional resources to improve heme production. ACLSm reaction is a sub-reaction of pyruvate dehydrogenase complex (PYRUVDEH-RXN), which converts pyruvate (pyr) (pyruvate) to acetyl-COA (accoa) (ACETYL-COA), which is the precursor of TCA cycle (GLYCOLYSIS-TCA-GLYOX-BYPASS) first step reaction. We believe that by decreasing this reaction flux rate we decrease CO2 (co2) (CARBON-DIOXIDE) production, slow down TCA cycle flux and thus decrease amino acids production, releasing additional resources for improved heme biosynthesis.

DHAD1m reaction is involved in L-valine biosynthesis (VALSYN-PWY) metabolism, Downregulation of DHAD1m reaction decreases also L-alanine (ala_L) (L-ALPHA-ALANINE) and L-leucine (leu_L) (LEU) biosynthesis precursors production, thus releasing additional resources for improved heme production.

**Aspartate 1-decarboxylase (PWY-5155)**

4.1.1.11 - aspartate 1-decarboxylase (ASP1DC) (ASPDECARBOX-RXN) (S6.12 ID 27).

ASP1DC reaction converts L-aspartate to L-alanine (ala_L) (L-ALPHA-ALANINE), thus reaction downregulation could release additional resources to increase intracellular heme production.

**Supplementary materials 5**

**Other reactions downregulation candidates reactions and their description.**

All suggested down-regulation reactions have ~ 17.0 – 17.3 % of step-weighted factor and have aproximatly the same effect on increased heme intracellular production. Exception is formate dehydrogenase with 16.14 step-weighted factor.

All reactions in the supplementary material will be described as:

Reaction name (ID in the model) (ID in the Metacyc) (Found in Supplementary material table)

All non – amino acids downregulation reactions are found in S6.11 supplementary materials

**Chitin synthase**

2.4.1.16 - chitin synthase (CHTNS) (CHITIN-SYNTHASE-RXN) (S6.11 ID 1).

Chitin is widely distributed in nature and is the second most abundant polysaccharide after cellulose. Also chitin is cell wall component of P. pastoris and is by-product of recombinant bioproduction processes ^4^. Decrease of chitin biosynthesis could reroute additional resources to heme production.

**Carnitine O-acetyltransferase**

2.3.1.7 - carnitine O-acetyltransferase (CSNATr) (CARNITINE-O-ACETYLTRANSFERASE-RXN) (S6.11 ID 2).

The carnitine O-acetyltransferase catalyses acetyl-CoA transfer to carnithine. It seems, that by decreasing this reaction- some acetyl-CoA (accoa) (ACETYL-COA) could be rerouted to heme synthesis.

**Dihydrofolate reductase**

1.5.1.3 - dihydrofolate reductase (DHFRim) (DIHYDROFOLATEREDUCT-RXN) (S6.11 ID 3).

This reaction is producing tetrahydrofolate (thf) (THF-GLU-N) from dihydrofolate (dhf) (DIHYDROFOLATE-GLU-N) – the folic acid derivative. Tetrahydrofolate (thf) (THF-GLU-N) is cofactor for many reactions especially in amino and nucleic acids biosynthesis ^5^. Decreasing its production rate additional resources could be redirected to heme production.

**Mannose-1-phosphate guanylyltransferase.**

2.7.7.22 - mannose-1-phosphate guanylyltransferase (MAN1PT2) (MANNPGUANYLTRANGDP-RXN) (S6.11 ID 4).

This reaction is producing GDP-D-mannose (gdpmann) (GDP-MANNOSE), which is required for cell wall in yeasts ^6^. Downregulation of this reaction would reroute additional resources for increased heme bioproduction.

**phosphatidylcholine-diacylglycerol acyltransferase**

2.3.1. - phosphatidylcholine-diacylglycerol acyltransferase (PCDAGAT) (RXN-12383) (S6.11 ID 5).

This reaction produces triglyceride (triglyc_SC) (Triacylglycerols).In the model there is average triglyceride reaction and is not included specific triglycerides. Metabolic model optimisations show that triglyceride production downregulation could release additional resources for improved heme production, but more detailed research for this specific downregulation candidate reaction must be done.

**Dolichyl-phosphate-mannose--protein mannosyltransferase**

2.4.1.109 – Dolichyl-phosphate-mannose--protein mannosyltransferase (dolpmmer) (2.4.1.109-RXN) (S6.11 ID 6).

This reaction is involved in O-glycosylation which is essential for yeast cell wall rigidity. Reactions downregulation could release additional resources to improve heme production. ^7^

**Glyoxylate and dicarboxylate metabolism**

1.1.1.37 - malate dehydrogenase (MDHp) (MALATE-DEH-RXN) (S6.11 ID 8);

1.2.1.2 (1.17.1.9) - formate dehydrogenase (FDH) (1.2.1.2-RXN) (S6.11 ID 9).

MDHp within the peroxisome converts malate (mal_L) (MAL) to oxaloacetate (oaa) (OXALACETIC_ACID). Simultaneously it seems, that oxaloacetate production in the cytoplasm is stimulated via pyruvate carboxylation (see section pyruvate). To our knowledge, there is no information on the particular need of malate dehydrogenase during methanol consumption within peroxisome; therefore, this reaction was found as good downregulation candidate.

FDH reaction is involved in many metabolic processes. In purine nucleobases degradation metabolim FDH converts formate (for) (FORMATE) to CO2 (co2) (CARBON-DIOXIDE). Downregulating FDH we could save carbon and help it to reroute it to heme production.

**Citric Acid Cycle (TCA) metabolism**

4.2.1.3 - Aconitate hydratase (ACONTm) (RXN-14047) (S6.11 ID 10);

2.3.3.1 - citrate synthase (CSm) (CITSYN-RXN) (S6.11 ID 11).

ACONTm and CSm reactions are involved in mitochondrial TCA cycle (PWY-5690) activity. TCA serves many functions - it is source for reduced cofactors (NADH and FADH) to donor proton for electron transport chain and synthesise ATP (atp) (ATP); as well as it has anabolic function to provide carbon backbone for amino acids – glutamine (gln_L) (GLN) to and glutamate (glu_L) (glutamate). Two CO2 (co2) (CARBON-DIOXIDE) is generated during full "turn" of TCA cycle ^8^. Downregulation of ACONTm and CSm would reduce TCA cycle (PWY-5690) activity, thus reducing its contribution in amino acid and energy production. CSm is the first reaction of the TCA cycle (PWY-5690) where citrate is synthesised from malate and acetyl group (provided by acetyl-coA (accoa) (ACETYL-COA)). The simultaneous downregulation of CSm together with downergulation of pyruvate decarboxylase complex (see section "pyruvate metabolism) abolish TCA (PWY-5690) activity, thus redirecting additional carbon for heme production.

**Phospholipid Metabolism.**

3.1.3.4 - diacylglycerol pyrophosphate phosphatase (DAGPYP_PP) (PHOSPHATIDATE-PHOSPHATASE-RXN) (S6.11 ID 12);

4.1.1.65 - phosphatidylserine decarboxylase (PSERDg_PP) (PHOSPHASERDECARB-RXN) (S6.11 ID 13);

2.3.1.23 - Lyso-phosphatidylcholine acyltransferase acyltransferase (LPCAT_PP) (2.3.1.23-RXN) (S6.11 ID 14).

DAGPYP_PP, PSERDg_PP, LPCAT_PP) reactions are involved in phospholipid metabolism: diacylglycerol and triacylglycerol biosynthesis (TRIGLSYN-PWY), phosphatidylserine (ps_SC) (L-1-PHOSPHATIDYL-SERINE) and phosphatidylethanolamine (pe_SC) (L-1-PHOSPHATIDYL-ETHANOLAMINE) biosynthesis (PWY-566). As in the model there is average triglyceride, fatty acid biosynthesis reaction thus specific, more detailed phospholipid reactions are not included in the model. Metabolic model optimisations show that downregulation of phospholipid metabolism could release additional resources to increase heme production, but more detailed research for this specific downregulation candidate reaction must be done.

**Pyruvate Metabolism**

1.2.4.1 - pyruvate dehydrogenase (PDHa2) (RXN0-1134) (S6.11 ID 15);

2.3.1.12 - pyruvate dehydrogenase (PDHbrm) (RXN0-1133) (S6.11 ID 16);

2.3.3.14 - homocitrate synthase (HCITSm) (HOMOCITRATE-SYNTHASE-RXN) (S6.11 ID 17);

2.3.1.9 (2.3.1.16) - acetyl-CoA C-acetyltransferase (ACACT1x) (ACETYL-COA-ACETYLTRANSFER-RXN) (S6.11 ID 18).

All these reactions take place in mitochondria and are catalysed by a proteins which collectively form one enzymatic unit - pyruvate dehydrogenase complex. As a sum - these reactions ensure enzymatic conversion of pyruvate (pyr) (pyruvate) to acetyl-coA (accoa) (ACETYL-COA) and CO2 (co2) (CARBON-DIOXIDE). If pyruvate decarboxylation is inhibited, pyruvate (pyr) (pyruvate) is redirected to formation of oxaloacetic acid (oaa) (OXALACETIC_ACID), which in turn can be used to amino acid synthesis (aspartate (asp_L) (L-ASPARTATE), threonine (thr_L) (THR), methionine (met_L) (MET)) ^9^ ; methionine (met_L) (MET) is necessary C1 metabolism. Excessive methionine synthesis would facilitate heme production.

ACACT1x reaction is involved in fatty acid oxidation in the mitochondria, mevalonate biosynthesis (mev_R) (MEVALONATE) and pyruvate (pyr) (PYRUVATE) fermentation. The reaction consumes acetyl-CoA (accoa) (ACETYL-COA). Downregulation of this reaction could "save" some acetyl-coA for intracellular heme production.

**Sphingolipid Metabolism**

1.14.13.169 ([EC 1.14.18.5](https://biocyc.org/META/NEW-IMAGE?type=REACTION&object=RXN-14250)) - Phytosphingosine synthesis (PSPHS) (RXN-14250) (S6.11 ID 19);

2.3.1.297 - Ceramide-2 synthase (CERS224er) (RXN-20399) (S6.11 ID 20);

2.3.1.297 - Ceramide-2 synthase (CERS226er) (RXN-20399) (S6.11 ID 21).

PSPHS, CERS224er and CERS226er reactions are involved in sphingolipid biosynthesis (SPHINGOLIPID-SYN-PWY). Sphingolipids are responsible for regulation of [cytoskeletal](https://en.wikipedia.org/wiki/Cytoskeletal) dynamics, the [cell cycle](https://en.wikipedia.org/wiki/Cell_cycle), [translation](https://en.wikipedia.org/wiki/Translation_(biology)), posttranslational protein modification, and the heat stress response ^10^. Reactions CERS224er and CER226er consume activated coA (coa) (CO-A): therefore by shutting down these reactions - additional acetyl-CoA might be rerouted for heme production.

**Sterol Metabolism**

2.7.1.36 - mevalonate kinase (MEVK4) (MEVALONATE-KINASE-RXN) (S6.11 ID 22);

4.1.3.5 (2.3.3.10) - Hydroxymethylglutaryl CoA synthase (HMGCOASm) (HYDROXYMETHYLGLUTARYL-COA-SYNTHASE-RXN) (S6.11 ID 23);

1.14.99.7 (1.14.14.17) – Squalene epoxidase (SQLErx) (SQUALENE-MONOOXYGENASE-RXN) (S6.11 ID 24);

1.14.19.41 - C-22 sterol desaturase (C22STDSx) (RXN3O-227) (S6.11 ID 25).

Sterol production is a proxy of biomass growth, thus decreasing production their production biomass synthesis would be hampered. On the other hand - growth coupled production model is aimed to increase heme production on the expense of biomass growth. Besides, desaturases are iron dependent enzymes ^11^ and thus they contribute to the exploitation of iron, which could be rerouted to heme synthesis.

**Pantothenate (PANTO-PWY) and CoA Biosynthesis (COA-PWY)**

1.1.1.169 - 2-dehydropantoate 2-reductase (DPRm) (2-DEHYDROPANTOATE-REDUCT-RXN) (S6.11 ID 26);

6.3.2.5 - phosphopantothenate-cysteine ligase (PPNCL2) (P-PANTOCYSLIG-RXN) (S6.11 ID 27).

DPRm and PPNCL2 reactions are involved in phosphopantothenate biosynthesis I (PANTO-PWY) and coenzyme A biosynthesis (COA-PWY) biochemical pathways accordingly, which in the end produces coa (coa) (CO-A) metabolite. Downregulation of 2 reactions is leading to decreased coa (coa) (CO-A) production and thus could free additional resources for improved heme production.

**Nucleotide Metabolism**

2.7.1.20 - adenosine kinase (ADNK1) (ADENOSINE-KINASE-RXN) (S6.11 ID 29);

6.3.4.2 - CTP synthase (CTPS1) (CTPSYN-RXN) (S6.11 ID 29);

1.1.1.205 – IMP dehydrogenase (IMPD) (IMP-DEHYDROG-RXN) (S6.11 ID 29);

1.17.4.2 - ribonucleoside-triphosphate reductase (RNTR2) (RXN-21263) (S6.11 ID 30);

1.17.4.2 - ribonucleoside-triphosphate reductase (RNTR4) (RXN-21265) (S6.11 ID 31);

2.7.4.12/2.7.4.13 - deoxyguanylate kinase (DGK1) (GMKALT-RXN) (S6.11 ID 32);

2.7.4.6 - nucleoside-diphosphate kinase (NDPK3) (CDPKIN-RXN) (S6.11 ID 33);

2.7.4.6 - nucleoside-diphosphate kinase (NDPK5) (DGDPKIN-RXN) (S6.11 ID 34);

2.7.4.4 / 2.7.4.9 – uridylate kinase (URIDK2r) (RXN-14220) (S6.11 ID 35);

1.17.4.2 - ribonucleoside-triphosphate reductase (RNTR1) (RXN-21262) (S6.11 ID 36);

1.17.4.1 - ribonucleoside-diphosphate reductase (RNDR4) (UDPREDUCT-RXN) (S6.11 ID 37).

The largest cluster of downregulation reactions are found in nucleotide metabolism.

To aim for lower growth speed - smaller flux share should be devoted the production of nucleotides and their salvage for the needs of DNA/ RNA synthesis. Therefore, the downregulation of nucleotide metabolism demonstrates options to downregulate growth rate and to simultaneously stimulate heme production yields.

Supplementary materials references

1. Castegna, A. *et al.* Identification and Functional Characterization of a Novel Mitochondrial Carrier for Citrate and Oxoglutarate in Saccharomyces cerevisiae. *J. Biol. Chem.* **285**, 17359–17370 (2010).

2. Huang, C.-W., Walker, M. E., Fedrizzi, B., Gardner, R. C. & Jiranek, V. Hydrogen sulfide and its roles in Saccharomyces cerevisiae in a winemaking context. *FEMS Yeast Res.* **17**, (2017).

3. Walvekar, A. S. & Laxman, S. Methionine at the Heart of Anabolism and Signaling: Perspectives From Budding Yeast. *Front. Microbiol.* **10**, (2019).

4. Zhang, X. *et al.* Enhanced bioproduction of chitin in engineered Pichia pastoris. *Food Biosci.* **47**, 101606 (2022).

5. Ducker, G. S. & Rabinowitz, J. D. One-Carbon Metabolism in Health and Disease. *Cell Metab.* **25**, 27–42 (2017).

6. YODA, K. *et al.* Defect in Cell Wall Integrity of the Yeast Saccharomyces cerevisiae Caused by a Mutation of the GDP-mannose Pyrophosphorylase Gene VIG9. *Biosci. Biotechnol. Biochem.* **64**, 1937–1941 (2000).

7. Gentzsch, M. & Tanner, W. The PMT gene family: protein O-glycosylation in Saccharomyces cerevisiae is vital. *EMBO J.* **15**, 5752–5759 (1996).

8. Malina, C., Larsson, C. & Nielsen, J. Yeast mitochondria: an overview of mitochondrial biology and the potential of mitochondrial systems biology. *FEMS Yeast Res.* **18**, (2018).

9. Sola, A., Maaheimo, H., Ylonen, K., Ferrer, P. & Szyperski, T. Amino acid biosynthesis and metabolic flux profiling of Pichia pastoris. *Eur. J. Biochem.* **271**, 2462–2470 (2004).

10. Cowart, L. A. & Obeid, L. M. Yeast sphingolipids: Recent developments in understanding biosynthesis, regulation, and function. *Biochim. Biophys. Acta - Mol. Cell Biol. Lipids* **1771**, 421–431 (2007).

11. Romero, A. M., Jordá, T., Rozès, N., Martínez-Pastor, M. T. & Puig, S. Regulation of yeast fatty acid desaturase in response to iron deficiency. *Biochim. Biophys. Acta - Mol. Cell Biol. Lipids* **1863**, 657–668 (2018).
